# Multiparametric Ultrasound in Chronic Viral Hepatitis: From Fibrosis and Portal Hypertension to Steatosis and Focal Lesion Characterisation

**DOI:** 10.3390/diagnostics16162502

**Published:** 2026-08-07

**Authors:** Krystian Mirowski, Andrzej Fedak, Jacek Czepiel, Jan Jamroś, Michal Kukla

**Affiliations:** 1 Clinical Department of Toxicology and Environmental Diseases, Jagiellonian University Medical College, 30-688 Krakow, Poland; 2 Department of Radiology, Jagiellonian University Medical College, 31-503 Krakow, Polandjan.jamros@student.uj.edu.pl (J.J.); 3 Clinical Department of Infectious Diseases, Jagiellonian University Medical College, 30-688 Krakow, Poland; jacek.czepiel@uj.edu.pl; 4 Clinical Department of Gastroenterology and Hepatology, Jagiellonian University Medical College, 30-688 Krakow, Poland

**Keywords:** multiparametric ultrasound, chronic hepatitis B, chronic hepatitis C, shear-wave elastography, contrast-enhanced ultrasound, liver fibrosis, portal hypertension, hepatocellular carcinoma, viral hepatitis elimination, non-invasive biomarkers

## Abstract

Chronic hepatitis B virus (HBV) and hepatitis C virus (HCV) infection remain leading causes of cirrhosis and hepatocellular carcinoma (HCC) worldwide, and continue to represent a major challenge despite the growing contribution of alcohol-related and metabolic dysfunction-associated steatotic liver disease. Direct-acting antivirals now cure most patients with chronic HCV infection, while nucleos(t)ide analogues durably suppress HBV replication, producing a rapidly expanding population of treated and cured patients. Many nonetheless retain residual fibrosis, portal hypertension and long-term cancer risk, creating a need for non-invasive long-term liver assessment. Multiparametric ultrasound (MPUS) integrates the evaluation of morphology, fibrosis, steatosis, haemodynamics and perfusion within a single examination. This narrative review summarises the evidence supporting MPUS in chronic viral hepatitis, covering elastographic fibrosis assessment, Baveno VII liver and spleen stiffness criteria for clinically significant portal hypertension, contrast-enhanced ultrasound characterisation of focal liver lesions, and emerging techniques such as microvascular and viscosity-sensitive imaging. Particular attention is given to the confounding effect of inflammatory activity on liver stiffness. MPUS is presented here as a proposed evaluation framework for organising complementary measurements within a single examination, and not as an approach of demonstrated clinical superiority: no comparative or outcome-based study has yet shown that acquiring these parameters together improves clinical decisions relative to the individual techniques applied according to current guidelines. Within this framework, the most plausible role of MPUS in the elimination era is longitudinal assessment of the treated liver, a proposition that requires prospective validation against clinical endpoints.

## 1. Introduction

Viral hepatitis remains one of the most important public health challenges worldwide. According to the World Health Organization (WHO), an estimated 240 million people were living with chronic hepatitis B virus (HBV) infection and 47 million with chronic hepatitis C virus (HCV) infection in 2024. Deaths attributable to viral hepatitis were estimated at approximately 1.3 million in 2024, the majority related to HBV infection [1]. In response, the WHO has adopted a strategy aiming to reduce new HBV and HCV infections by 90% and hepatitis-related mortality by 65% by 2030 compared with 2015 levels [2,3].

The prospects for achieving these goals have improved substantially owing to major advances in antiviral therapy. Direct-acting antivirals (DAAs), effective across all major HCV genotypes, can cure the vast majority of patients with chronic HCV infection, with sustained virological response rates exceeding 95% [4]. In HBV infection, nucleos(t)ide analogues provide durable suppression of viral replication and slow disease progression, while vaccination programmes have markedly reduced the incidence of new infections [5]. As a result, the population of treated and cured patients continues to grow. Nevertheless, successful viral control does not eliminate risk completely. In patients with advanced fibrosis or cirrhosis, the risk of hepatic decompensation and hepatocellular carcinoma (HCC) may persist for many years after viral suppression or eradication [6,7,8].

Monitoring this expanding population represents a major clinical challenge. Although liver biopsy served for many years as a reference method for fibrosis assessment, its limitations, including invasiveness, sampling error and interobserver variability, restrict its role in long-term follow-up. Today, liver biopsy is generally reserved for selected diagnostic situations, while non-invasive methods form the basis of disease assessment and monitoring [9,10,11].

Among these, ultrasound-based techniques occupy a prominent position. Their main advantages include wide availability, low cost and the absence of exposure to ionising radiation. These features make ultrasound particularly suitable for repeated examinations and long-term patient monitoring. Over the past two decades, liver ultrasound has evolved from a largely qualitative imaging modality into a quantitative and multiparametric diagnostic tool [9,12,13,14].

Modern ultrasound provides information not only on liver morphology but also on tissue mechanical properties, hepatic fat content, haemodynamic alterations associated with portal hypertension and tissue perfusion [13,14,15,16,17,18,19]. The development of these techniques has established ultrasound as one of the most important non-invasive tools for the diagnosis and follow-up of chronic liver diseases [9,13,14].

At the same time, the understanding of chronic liver disease has evolved. Fibrosis, long regarded as the principal imaging target, is now recognised as a relatively late manifestation of disease progression. Earlier events include necroinflammatory activity, liver sinusoidal endothelial cell (LSEC) dysfunction and microvascular remodelling [20,21,22,23,24]. These processes influence both the mechanical and functional properties of the liver and are increasingly accessible through advanced imaging techniques [23,24,25,26,27,28].

In this review, we discuss the role of multiparametric ultrasound (MPUS) in chronic viral hepatitis. Particular attention is given to the assessment of fibrosis, portal hypertension, steatosis and focal liver lesions, as well as to emerging approaches for evaluating microvascular changes, perfusion and tissue viscosity [13,15,19,26,27,28,29,30]. We review both well-established clinical applications and techniques that remain under investigation.

## 2. The Concept of Multiparametric Ultrasound

**Figure 1:**
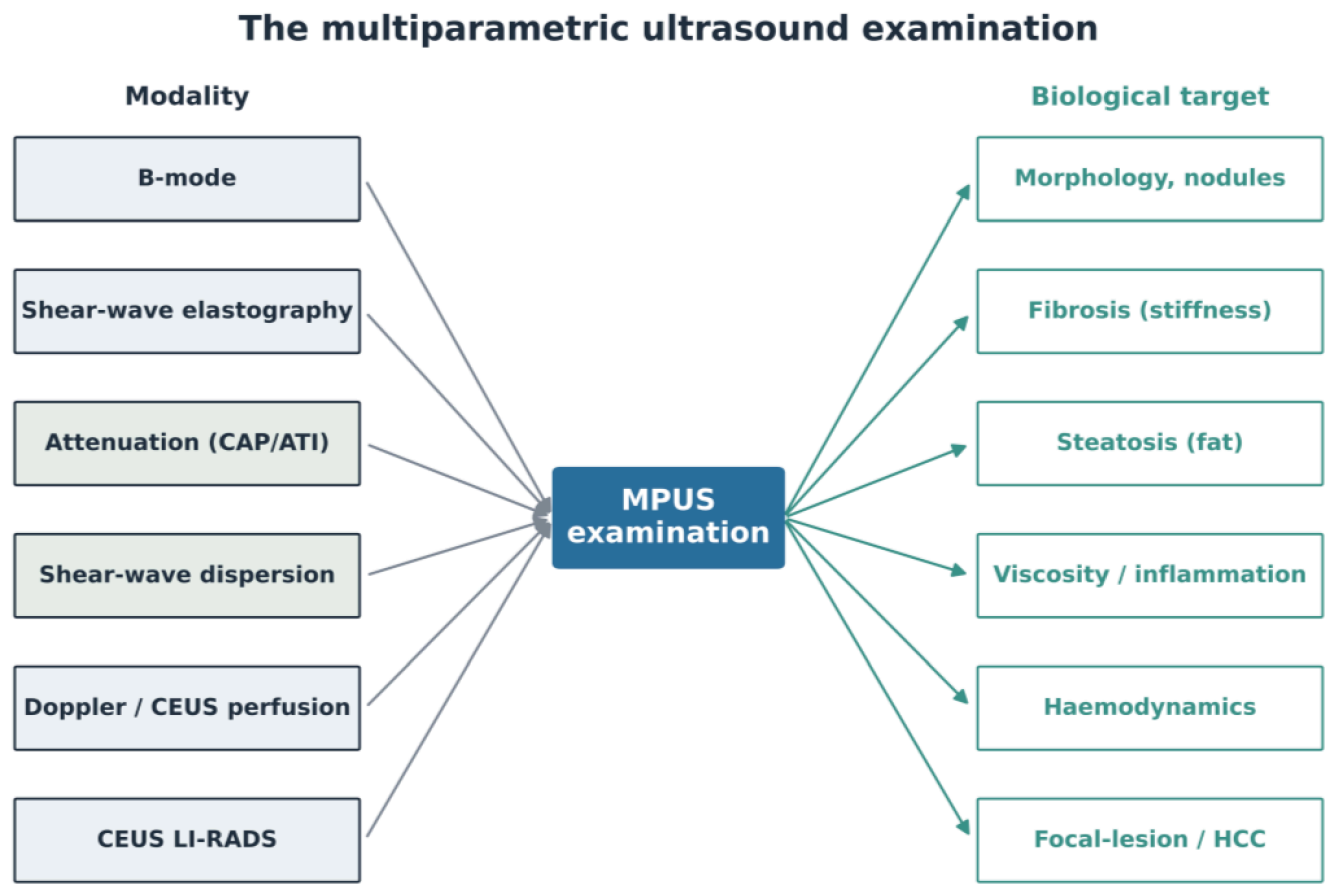
The multiparametric ultrasound examination in chronic viral hepatitis. A single examination integrates B-mode morphology, shear-wave elastography (fibrosis), attenuation imaging (steatosis), shear-wave dispersion (viscosity and inflammation), Doppler and contrast-enhanced ultrasound (haemodynamics and perfusion) and CEUS LI-RADS (focal-lesion characterisation), each probing a distinct biological property of the diseased liver.

Multiparametric ultrasound (MPUS) combines several complementary imaging techniques that reflect different aspects of chronic liver disease biology. By integrating the assessment of morphology, tissue mechanical properties, steatosis, haemodynamics and perfusion, MPUS provides a more comprehensive characterisation of the diseased liver than any individual technique alone [12,13,14]. The following sections review the diagnostic and prognostic value of individual MPUS components in patients with chronic viral hepatitis (Figure 1).

Two levels of claim should be distinguished at the outset. The individual components of MPUS have been validated separately, and several are recommended in current international documents for defined clinical questions [9,10,13,14,15,19]. By contrast, the proposition that acquiring them within a single examination improves patient outcomes or clinical decisions has not been tested in comparative or outcome-based studies. MPUS is therefore used in this review as a proposed evaluation framework, that is, a way of organising complementary measurements and interpreting them jointly, rather than as a modality with proven superiority over its individual components. Statements about the added value of integration should be read as hypotheses requiring prospective validation.

## 3. Materials and Methods

This article was designed as a narrative review of the role of multiparametric ultrasound (MPUS) in chronic viral hepatitis, with particular emphasis on fibrosis assessment, portal hypertension, hepatic haemodynamics, microvascular alterations and emerging imaging biomarkers in the context of long-term monitoring after antiviral therapy.

Relevant publications published between January 2000 and April 2026 were identified through searches of PubMed/MEDLINE, Scopus and Google Scholar (last search 30 April 2026), restricted to English-language publications, using combinations of keywords related to chronic hepatitis B, chronic hepatitis C, liver fibrosis, cirrhosis, transient elastography, shear-wave elastography, acoustic radiation force impulse imaging, controlled attenuation parameter, attenuation imaging, contrast-enhanced ultrasound (CEUS), portal hypertension, spleen stiffness, hepatocellular carcinoma surveillance, shear-wave dispersion and multiparametric ultrasound. Seminal studies published before this period were additionally included where necessary to provide historical context and to illustrate key developments in liver pathophysiology, hepatic vascular biology and ultrasound imaging.

Priority was given to original research articles, meta-analyses, clinical practice guidelines, consensus statements and influential review articles. Where several sources addressed the same question, priority was given in the following order: current international guidelines and consensus documents; meta-analyses and individual-patient-data analyses; prospective studies using histological, haemodynamic or clinical reference standards; and finally single-centre or exploratory studies, which are cited only to illustrate techniques that remain investigational. Particular attention was paid to studies addressing fibrosis staging, steatosis quantification, portal hypertension, vascular and perfusion imaging, focal liver lesion characterisation, and the role of ultrasound-based biomarkers in the management of chronic viral liver disease.

As this was a narrative review, no formal systematic-review protocol, PRISMA-based study selection process or quantitative meta-analysis was performed. A representative PubMed search combined terms such as (“chronic hepatitis B” OR “chronic hepatitis C”) AND (“transient elastography” OR “shear wave elastography” OR “contrast-enhanced ultrasound” OR “multiparametric ultrasound”). Screening and final selection of references were carried out by two authors (K.M. and A.F.), with disagreements resolved by discussion with the senior author (M.K.). After removal of duplicates identified across databases and screening of titles and abstracts, the remaining full-text publications were assessed, and the references cited in this article were retained as the primary supporting evidence. Publications were selected on the basis of their scientific relevance and contribution to the understanding of the biological and clinical foundations of MPUS; no formal critical appraisal of methodological quality or risk-of-bias assessment was undertaken. Diagnostic performance measures reported in this review are presented within the context of the original studies and should not be extrapolated beyond the populations in which they were obtained.

Accordingly, this review should be regarded as an integrative synthesis of the available evidence, aimed at linking the biological mechanisms of chronic viral liver disease with contemporary ultrasound-based diagnostic approaches and their potential role in the era of viral hepatitis elimination.

## 4. An Integrated Approach to Liver Assessment

The following sections review the individual components of multiparametric ultrasound and their role in the assessment and long-term monitoring of patients with chronic viral hepatitis. Although each technique provides distinct diagnostic information, their greatest clinical value lies in their combined interpretation within a single examination. This integrated approach enables a more comprehensive assessment of liver structure, function and haemodynamics than any individual parameter alone (Table 1).

## 5. Elastographic Assessment of Liver Fibrosis

Assessment of liver fibrosis by elastography represents the most extensively validated non-invasive application of ultrasound in chronic viral hepatitis and remains the cornerstone of multiparametric liver evaluation [9,10,13]. Vibration-controlled transient elastography (VCTE) was the first elastographic technique to gain widespread clinical acceptance and remains the most extensively validated method for non-invasive fibrosis assessment. Over the past two decades, it has become firmly established in routine hepatology practice and has been incorporated into major international guidelines [9,10,13].

More recently, ultrasound systems integrating conventional B-mode imaging with elastographic assessment have expanded the diagnostic capabilities of liver ultrasound. These include point shear-wave elastography (pSWE), based on acoustic radiation force impulse (ARFI) technology, and two-dimensional shear-wave elastography (2D-SWE). Unlike conventional VCTE, these techniques allow stiffness measurements to be performed under direct B-mode guidance, enabling simultaneous assessment of liver morphology and targeted placement of the measurement region [9,13,31,32,33]. Although newer transient elastography platforms, such as Hepatus and FibroTouch (both Wuxi Hisky Medical Technologies, Wuxi, China), also incorporate B-mode imaging, shear-wave elastography remains more seamlessly integrated into comprehensive ultrasound examinations and multiparametric liver assessment [13].

All of these techniques provide quantitative estimates of liver stiffness expressed in kilopascals (kPa) or metres per second (m/s), and their acquisition and interpretation have been standardised by successive guidelines from EFSUMB, WFUMB and the Society of Radiologists in Ultrasound [9,10,13].

The clinical relevance of elastography stems from the central role of fibrosis in the natural history of chronic viral hepatitis. Histologically, fibrosis is commonly staged using the METAVIR scoring system, ranging from F0 (no fibrosis) to F4 (cirrhosis), while inflammatory activity is graded separately from A0 to A3. Numerous studies have demonstrated a close association between liver stiffness and fibrosis stage, establishing elastography as a reliable surrogate marker of hepatic fibrotic burden [31,32,33,34].

Among available techniques, the strongest evidence base derives from patients with chronic HBV and HCV infection, in whom elastography was initially validated against histological assessment. In chronic hepatitis B, a meta-analysis of VCTE reported pooled AUROC values of approximately 0.86 for significant fibrosis, 0.89 for advanced fibrosis and 0.93 for cirrhosis, with optimal cut-off values around 7.9, 8.8 and 11.7 kPa, respectively, although thresholds varied according to inflammatory activity [34]. Comparable results have been reported for pSWE and 2D-SWE. An individual-patient-data meta-analysis demonstrated AUROC values of approximately 0.86 for significant fibrosis and 0.93 for cirrhosis in HCV, and 0.91 and 0.96, respectively, in HBV, with the greatest advantage of 2D-SWE observed in HBV cohorts [31]. Meta-analyses of pSWE have likewise shown high diagnostic accuracy, with pooled AUROC values increasing from approximately 0.84 for significant fibrosis to 0.91 for cirrhosis (Table 2) [32,33].

These findings support the use of elastography both for identifying advanced fibrosis and cirrhosis and for excluding clinically significant liver disease in low-risk patients. Consequently, elastography has become an integral component of routine assessment and long-term follow-up in chronic viral hepatitis [9,10,13].

Importantly, liver stiffness reflects the overall mechanical properties of the liver rather than fibrosis alone, and is influenced by congestion, cholestasis and, above all in chronic viral hepatitis, necroinflammatory activity [20,21,22]. This relationship is examined in the following section.

## 6. Liver Stiffness Beyond Fibrosis

A key concept in elastography is that liver stiffness should not be equated with fibrosis. Elastography measures a biomechanical property of the liver, whereas fibrosis represents only one of several factors contributing to that property. While extracellular matrix deposition and architectural distortion are the principal determinants of increased stiffness in chronic liver disease, necroinflammatory activity, hepatocellular swelling, oedema, cholestasis, vascular congestion and alterations in sinusoidal pressure may also influence elastographic measurements [20,21,22,23]. Accordingly, liver stiffness is best regarded as an integrated biomarker reflecting both structural and functional aspects of liver disease [22].

The mechanisms responsible for increased liver stiffness extend beyond collagen accumulation alone. Fibrosis increases stiffness through progressive deposition and cross-linking of extracellular matrix proteins, formation of fibrous septa and distortion of the normal hepatic architecture. However, active inflammation may also substantially alter tissue mechanics. Hepatocellular injury leads to cellular swelling, inflammatory cell infiltration and interstitial oedema, all of which increase tissue resistance to shear-wave propagation. At the same time, inflammatory mediators promote sinusoidal vasoconstriction and microvascular dysfunction, increasing intrahepatic vascular resistance and tissue pressure [20,21,22,23,24]. Consequently, liver stiffness reflects not only the amount of scar tissue present but also the biological activity of the disease.

Importantly, this distinction is reflected in contemporary hepatology guidelines, which formulate clinical recommendations in terms of fibrosis stage, cirrhosis, clinically significant portal hypertension and liver-related outcomes rather than liver stiffness itself [15]. Clinical decisions are not made because a patient has a particular stiffness value; they are made because that value is interpreted as a marker of advanced fibrosis, cirrhosis or portal hypertension. Liver stiffness should therefore be viewed as a surrogate biomarker rather than the biological process of interest itself.

**Figure 2:**
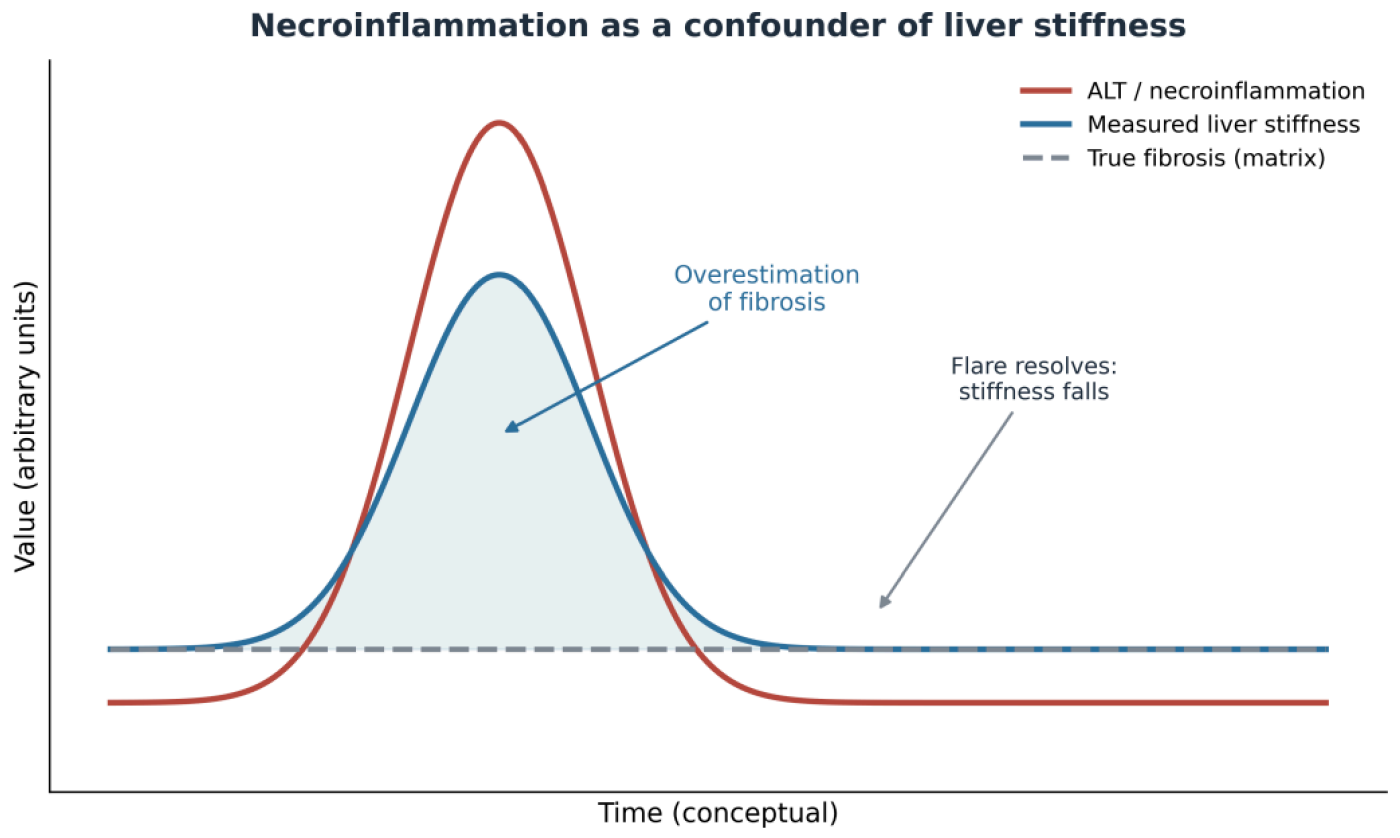
Necroinflammation as the central confounder of liver stiffness. Active hepatitis and aminotransferase flares raise measured stiffness independently of fibrosis stage and then fall as activity resolves, so that stiffness obtained during active disease overestimates fibrosis. The same inflammatory signal motivates viscosity- and perfusion-based imaging designed to capture biologically active disease. Curves are conceptual and not derived from measured data.

This distinction is particularly important in chronic viral hepatitis, where inflammation and stiffness are closely linked. Acute and active hepatitis can raise stiffness values markedly and reversibly, irrespective of the underlying fibrosis stage. In acute viral hepatitis, transient elastography values may reach the cirrhotic range at the peak of aminotransferase elevation and subsequently decline as the inflammatory flare resolves (Figure 2), demonstrating that a single elevated stiffness measurement cannot be equated with established fibrosis [20]. Similar observations have been reported in chronic viral hepatitis, where liver stiffness correlates with alanine aminotransferase levels and may change substantially in parallel with fluctuations in inflammatory activity [21,22].

The dynamic component of stiffness is further illustrated by the rapid decline in liver stiffness frequently observed after successful antiviral therapy. Reductions occurring within months of viral suppression or eradication are often too large and too rapid to be explained solely by regression of fibrosis, which generally evolves over years. Instead, they are thought to reflect resolution of necroinflammatory activity, improvement in sinusoidal function and changes in intrahepatic haemodynamics. Long-term decreases in stiffness likely represent a combination of these reversible factors and genuine fibrosis regression [35,36].

These observations have important clinical implications. For fibrosis staging, elastographic measurements should ideally be obtained when disease activity is stable, and interpretation should take into account disease aetiology and biochemical activity. Failure to do so may result in overestimation of fibrosis severity. Other factors, including hepatic congestion, cholestasis, severe steatosis and the postprandial state, may influence stiffness measurements in a similar manner and should also be considered [22]. In the case of severe steatosis, both the direction and the magnitude of the effect remain uncertain: hepatic fat is closely associated with obesity, insulin resistance and necroinflammatory activity, and also increases the proportion of technically unreliable acquisitions, so that its independent contribution to measured stiffness is difficult to isolate [17,18,22].

At the same time, the sensitivity of liver stiffness to inflammation highlights a biologically meaningful signal rather than merely a diagnostic limitation. If inflammatory activity alters the mechanical and haemodynamic properties of the liver, techniques designed to assess tissue viscosity and perfusion may provide complementary information that cannot be captured by stiffness measurements alone [23,24,28]. This concept forms an important link between established elastographic methods and newer approaches, such as shear-wave dispersion and advanced perfusion imaging, discussed in later sections of this review.

**Figure 3:**
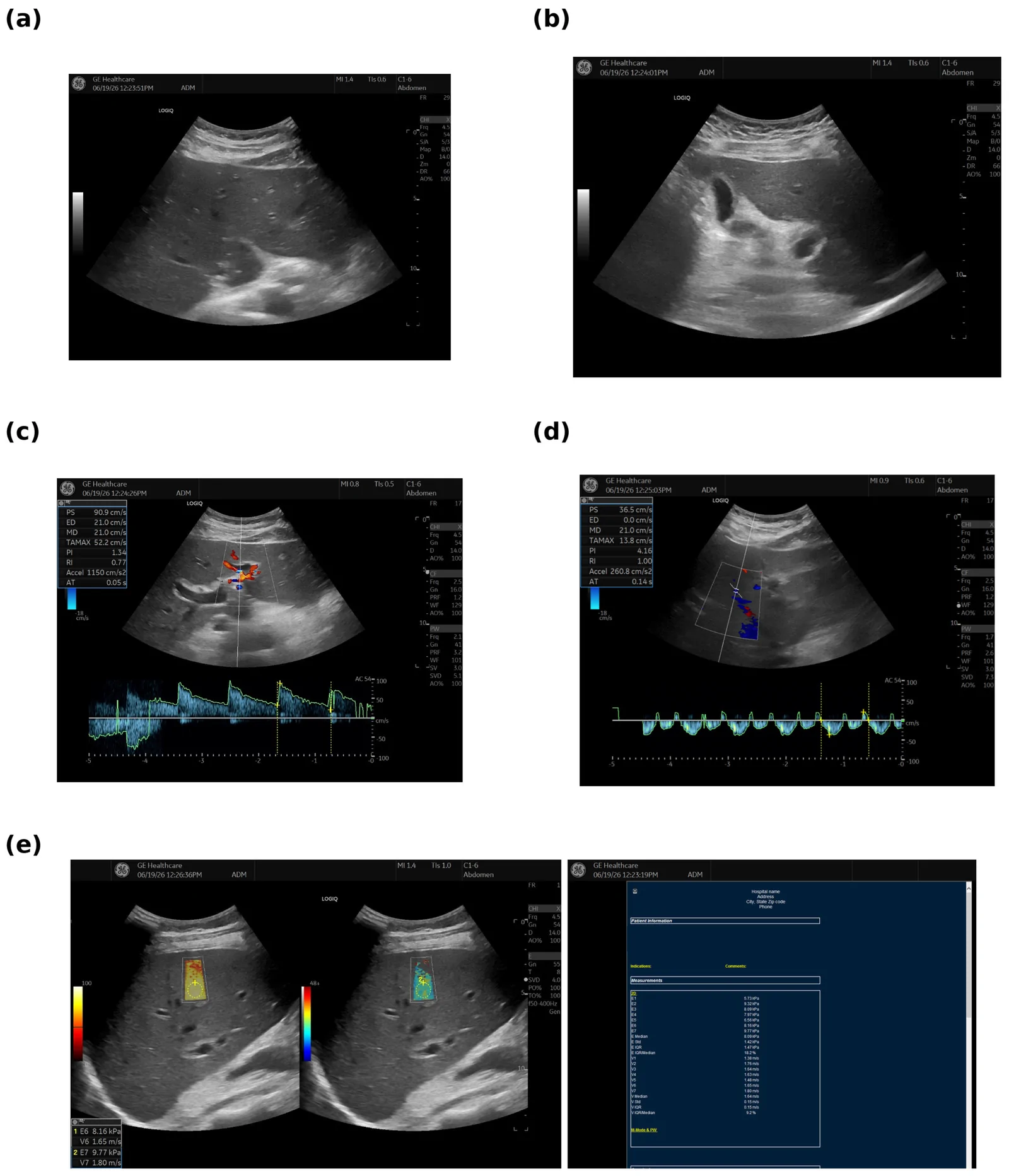
Ultrasound findings in acute hepatotropic viral injury, illustrated in a patient with acute hepatitis A. (**a**) B-mode image showing the starry-sky appearance, with prominent echogenic portal venous walls against a hypoechoic parenchyma. (**b**) Gallbladder wall thickening. (**c**) Spectral Doppler of the hepatic artery with a resistive index of 0.77. (**d**) Spectral Doppler of the hepatic vein showing increased pulsatility. (**e**) Two-dimensional shear-wave elastography with the corresponding measurement detail, showing elevated liver stiffness recorded during the acute phase. All findings are non-specific: they may occur in other forms of acute hepatotropic viral injury, while the Doppler indices are additionally influenced by cardiac and respiratory factors and by the acquisition protocol. The panels are single acquisitions obtained during the acute episode; serial measurements would be required to establish that the increase in stiffness is reversible. In (**c**) and (**d**), the yellow dotted lines and cross marks are the machine-generated callipers from which the displayed spectral Doppler indices were derived, and negative values on the velocity scale denote flow directed away from the transducer. In (**e**), the colour overlays are the two-dimensional shear-wave elastography maps displayed on the system’s own scales, the yellow cross and dashed circle mark the measurement region of interest, and the adjacent panel lists the individual stiffness measurements obtained during the acquisition. All annotations and scales are generated by the ultrasound system and the images are reproduced without modification.

**Figure 4:**
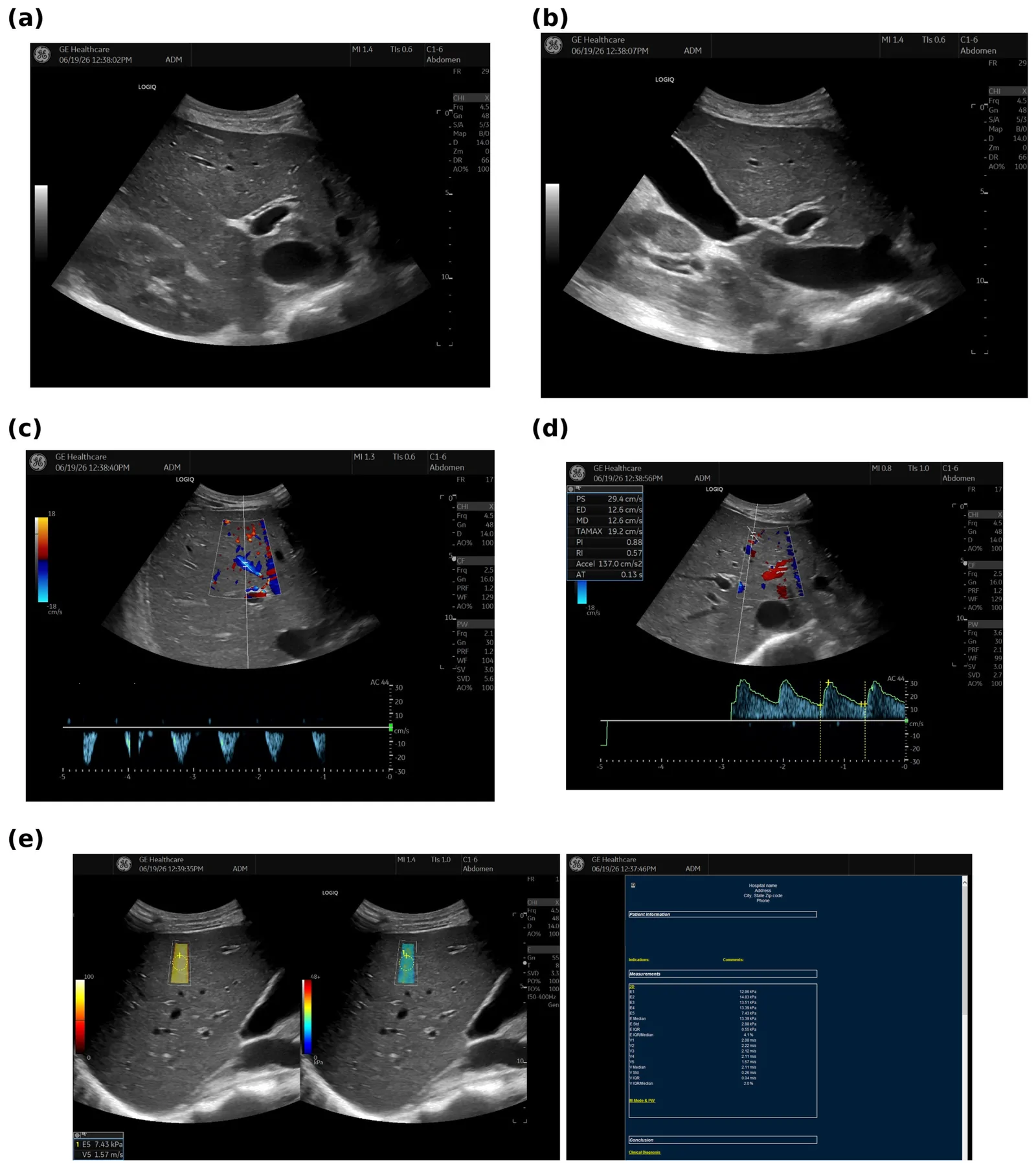
Ultrasound findings in chronic hepatitis C. (**a**) B-mode image showing mildly heterogeneous hepatic parenchymal echotexture. (**b**) Gallbladder wall thickening with periportal oedema. (**c**) Doppler assessment of the hepatic vein showing phasic flow variation related to the cardiac cycle. (**d**) Spectral Doppler of the hepatic artery showing normal antegrade flow and a normal resistive index (RI = 0.57). (**e**) Two-dimensional shear-wave elastography with the corresponding measurement detail, showing heterogeneous liver stiffness (median 13.4 kPa); stiffness heterogeneity should not be equated with a specific fibrosis distribution. In (**c**) and (**d**), the yellow dotted lines and cross marks are the machine-generated callipers from which the displayed spectral Doppler indices were derived, and negative values on the velocity scale denote flow directed away from the transducer. In (**e**), the colour overlays are the two-dimensional shear-wave elastography maps displayed on the system’s own scales, the yellow cross and dashed circle mark the measurement region of interest, and the adjacent panel lists the individual stiffness measurements obtained during the acquisition. All annotations and scales are generated by the ultrasound system and the images are reproduced without modification.

The imaging series presented in this review illustrates this principle. Acute hepatitis A is shown here purely as a model of intense but reversible necroinflammation: the accompanying ultrasound findings, including the starry-sky appearance (Figure 3a), gallbladder wall thickening (Figure 3b), altered hepatic arterial (Figure 3c) and hepatic venous (Figure 3d) Doppler signals and markedly increased shear-wave stiffness measured during the acute phase (Figure 3e), reflect active inflammation rather than established fibrosis [20]. These images are included solely as a conceptual illustration of the influence of necroinflammation on stiffness and fall outside the chronic-hepatitis focus of this review. They are single acquisitions, so the reversibility of the observed changes cannot be demonstrated here and is inferred from published serial measurements in acute viral hepatitis [20]. The findings are also not specific to hepatitis A and may be encountered in other forms of acute hepatotropic viral injury, including acute hepatitis E, acute exacerbations of chronic hepatitis B and Epstein–Barr virus or cytomegalovirus hepatitis; the series should therefore be read as a general model of acute viral liver injury. By contrast, the chronic hepatitis C series demonstrates the heterogeneous parenchymal echotexture (Figure 4a), gallbladder wall thickening with periportal oedema (Figure 4b), phasic hepatic venous flow (Figure 4c), a normal hepatic arterial resistive index (Figure 4d) and heterogeneous liver stiffness (Figure 4e) that characterise established viral liver disease. Together, these examples emphasise that an elevated stiffness value must always be interpreted in the context of disease activity and aetiology.

## 7. Longitudinal Assessment Before and After Antiviral Therapy

The emergence of a large population of successfully treated patients is one of the defining consequences of the viral hepatitis elimination era. This shift has increased the importance of longitudinal assessment, extending from baseline evaluation before antiviral therapy to long-term monitoring after viral suppression or eradication. Following treatment, liver stiffness commonly declines, often substantially. In chronic hepatitis C, a systematic review and meta-analysis demonstrated significant reductions in liver stiffness after treatment, with the largest and most rapid decreases observed after interferon-free direct-acting antiviral regimens achieving sustained virological response (SVR) [35]. Similar findings have been reported in chronic hepatitis B, where long-term nucleos(t)ide analogue therapy results in progressive decreases in liver stiffness that parallel biochemical improvement and favourable histological changes [36].

The interpretation of these changes requires caution. As discussed in Section 6, much of the early decline reflects resolution of necroinflammatory activity and improvement in sinusoidal function rather than regression of extracellular matrix, so that a normalised stiffness value shortly after treatment should not be interpreted as proof of fibrosis reversal [21,35].

The biology underlying these changes differs between the two infections, and post-treatment stiffness should be interpreted accordingly. In chronic hepatitis C, sustained virological response represents eradication of the virus, so the necroinflammatory stimulus is removed and the subsequent trajectory of liver stiffness reflects resolution of inflammation followed, in some patients, by slow matrix regression. In chronic hepatitis B, nucleos(t)ide analogue therapy achieves durable suppression of viral replication rather than cure: covalently closed circular DNA persists in the hepatocyte nucleus, viral DNA integrated into the host genome is not removed, functional cure with loss of HBsAg is uncommon, and low-grade necroinflammatory activity may continue or recur, particularly after treatment interruption [5,36]. A decline in liver stiffness during nucleos(t)ide analogue therapy therefore documents control of inflammatory activity rather than removal of the underlying oncogenic driver, and, unlike in cured HCV infection, the biological stimulus for disease progression is suppressed rather than eliminated.

These observations highlight an often overlooked principle: meaningful post-treatment assessment begins before antiviral therapy is initiated. Where feasible, baseline evaluation ideally includes not only liver stiffness measurement (LSM) but also a structured assessment of the portal venous system, spleen size, signs of portal hypertension and, where available, spleen stiffness measurement (SSM) [15,16,37]. Such parameters define the severity of liver disease at treatment initiation and establish the reference framework against which subsequent changes can be interpreted.

This baseline assessment is particularly important because liver stiffness may decline rapidly after viral suppression or eradication, whereas portal hypertension and its consequences often regress more slowly and may persist despite marked improvements in liver stiffness. Without pre-treatment documentation of liver and spleen stiffness and portal haemodynamics, interpretation of post-treatment findings becomes substantially more difficult. In this regard, multiparametric ultrasound offers a unique advantage by allowing simultaneous assessment of liver morphology, fibrosis burden, portal circulation and splenic involvement within a single examination [12,13,15].

Interpretation of post-treatment liver stiffness should therefore be complemented by assessment of the spleen and portal circulation. In contrast to liver stiffness, which is strongly influenced by necroinflammatory activity, spleen stiffness appears to reflect portal hypertension more directly and may remain elevated despite virological control [15,16,37]. Combined evaluation of liver stiffness measurement (LSM) and spleen stiffness measurement (SSM), as incorporated into the Baveno VII framework, may therefore provide a more robust assessment of residual portal hypertension and long-term clinical risk than liver stiffness alone [15,37].

The need for careful longitudinal assessment is underscored by the fact that viral cure or suppression does not eliminate future risk. Patients with advanced fibrosis or cirrhosis before treatment remain at increased risk of hepatocellular carcinoma (HCC), clinically significant portal hypertension and hepatic decompensation for many years after viral control [6,7,8]. Importantly, these risks are determined primarily by the severity of pre-existing liver damage rather than by post-treatment viral status alone.

**Figure 5:**
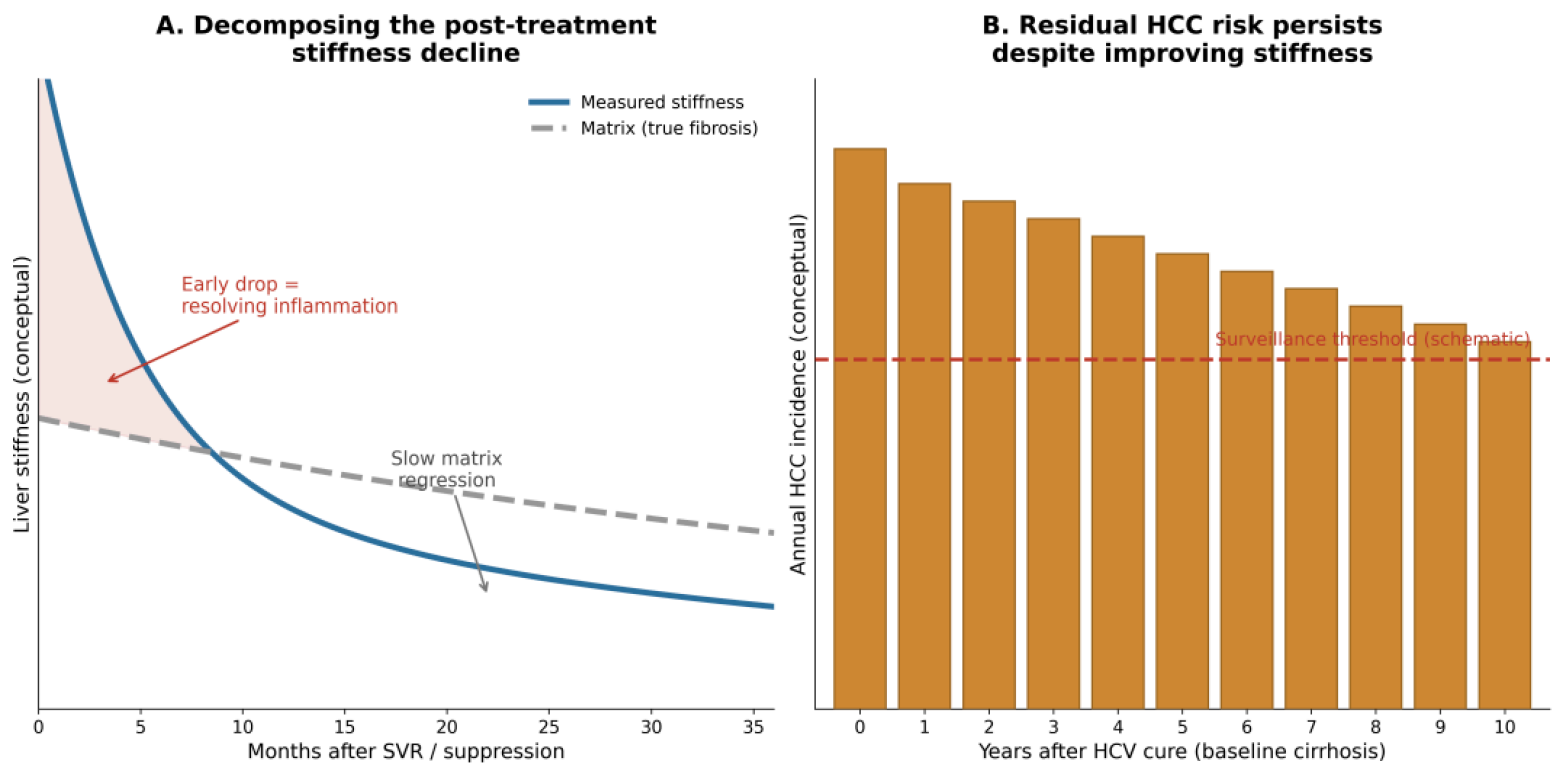
Imaging the treated liver. (**A**) After sustained virological response or viral suppression, liver stiffness declines; the early reduction reflects resolving necroinflammation more than true matrix regression. (**B**) Residual risk of hepatocellular carcinoma persists in patients with advanced baseline disease, so improving stiffness does not justify discontinuing surveillance. Panels are conceptual illustrations and do not represent data from any specific study; the indicated values and thresholds are schematic only.

Accordingly, the principal goal of imaging after antiviral therapy is not merely to document improvement in liver stiffness but to characterise residual liver disease and identify patients who require continued surveillance. In this setting, a multiparametric approach is particularly attractive. Interpretation of liver stiffness alongside portal haemodynamics, spleen-related parameters, steatosis assessment and, where indicated, contrast-enhanced imaging provides a more complete picture of the treated liver than any individual biomarker alone [12,13,15,19]. We would therefore propose, as a hypothesis rather than an established finding, that the principal contribution of MPUS lies less in diagnosing advanced disease than in monitoring its evolution after successful antiviral therapy in the growing population of treated patients, in whom the early post-treatment decline in liver stiffness reflects resolving necroinflammation rather than matrix regression (Figure 5A) while the residual risk of hepatocellular carcinoma persists in those with advanced baseline disease (Figure 5B). This proposal is not supported by direct comparative or outcomes-based evidence, and the combination of several ultrasound parameters should not at present be used to modify surveillance intervals or to reclassify individual HCC risk; such decisions should continue to follow current guideline criteria.

## 8. Haemodynamic Assessment and Portal Hypertension

Beyond fibrosis staging, multiparametric ultrasound addresses the haemodynamic consequence of chronic liver disease that most strongly determines clinical outcome: portal hypertension. While fibrosis reflects structural liver damage, portal hypertension represents its functional expression and is directly linked to the development of oesophageal varices, ascites, hepatic decompensation and liver-related mortality [15,16,37]. For this reason, contemporary hepatology increasingly focuses not only on fibrosis staging but also on identifying patients with clinically significant portal hypertension (CSPH) [15].

The Baveno VII consensus formalised the concept of compensated advanced chronic liver disease (cACLD) and provided pragmatic, stiffness-based rules for CSPH assessment. Within this framework, a liver stiffness measurement (LSM) of 25 kPa or greater is sufficient to rule in CSPH in patients with cACLD of viral or alcohol-related aetiology, while values of 15 kPa or below combined with a platelet count of at least 150 × 10^9^/L make CSPH unlikely (Table 3) [15]. These thresholds were derived predominantly with vibration-controlled transient elastography in cohorts with compensated advanced chronic liver disease of viral or alcohol-related aetiology, and the corresponding spleen-stiffness thresholds of 21 and 50 kPa were established with a dedicated 100 Hz spleen module in comparable populations [15,16,37,38]. Their performance cannot be assumed to be identical in other settings. After HCV cure, liver stiffness falls more rapidly than portal pressure, so post-treatment values may underestimate residual portal hypertension; in decompensated disease the Baveno VII rules, which were developed for compensated patients, do not apply; and in patients with superimposed metabolic or alcohol-related liver disease, or with ongoing necroinflammatory activity, both liver and spleen stiffness may be elevated independently of portal pressure [15,35,36,37,38]. A substantial proportion of patients nevertheless remain within an indeterminate range between these thresholds.

Spleen stiffness measurement (SSM) helps reduce this diagnostic grey zone. In advanced chronic liver disease, the spleen reflects portal pressure more directly than the liver itself, and spleen stiffness has been shown to correlate with the presence of portal hypertension, oesophageal varices and clinical outcomes in viral cirrhosis [16,37]. Accordingly, Baveno VII endorses spleen stiffness assessment to help rule in and rule out CSPH in patients with cACLD due to viral hepatitis. Combining liver and spleen stiffness reduces the number of indeterminate patients and improves risk stratification compared with liver stiffness assessment alone [15,37,38]. In routine practice, the platelet count is the weakest element of these rules, because thrombocytopenia in chronic viral hepatitis is frequently multifactorial: direct marrow suppression by HBV or HCV, previous interferon exposure, alcohol use, immune-mediated platelet destruction, nutritional deficiency and coexisting haematological disorders may all lower the platelet count independently of portal pressure, while splenic sequestration reflects portal hypertension only in part. In a patient with thrombocytopenia whose liver stiffness lies within the indeterminate range, spleen stiffness measurement provides a more direct haemodynamic correlate of portal pressure and can help establish whether the low platelet count reflects clinically significant portal hypertension or another mechanism [15,16,37,38].

From an imaging perspective, the Baveno VII framework can be viewed as an integral component of a contemporary ultrasound examination. Although commonly discussed in terms of numerical thresholds, its practical implementation increasingly relies on ultrasound-derived biomarkers. Liver stiffness measurement, spleen stiffness assessment, spleen size and Doppler evaluation of the portal venous system provide complementary information regarding the presence and severity of portal hypertension [15,37]. Rather than representing isolated measurements, these parameters collectively contribute to a haemodynamic phenotype of advanced chronic liver disease. In this sense, multiparametric ultrasound provides a practical platform for implementing Baveno VII recommendations within a single, non-invasive examination [12,13,15].

Importantly, portal hypertension is not solely a consequence of fibrosis. Dynamic changes in vascular tone, sinusoidal endothelial dysfunction and intrahepatic microvascular remodelling contribute substantially to increased portal resistance [23,24]. This observation again illustrates the distinction between fibrosis and liver function discussed in previous sections. Two patients with similar fibrosis stages may exhibit markedly different portal pressures and clinical risk profiles.

Within a multiparametric ultrasound examination, elastographic assessment can be complemented by Doppler evaluation of the portal venous system. Measurements of portal vein diameter and flow velocity, splenic vein characteristics, collateral circulation and signs of portal–systemic shunting provide additional information regarding the haemodynamic consequences of chronic liver disease [15,37]. Although Doppler findings alone are generally insufficient to diagnose CSPH, they contribute valuable contextual information when interpreted alongside liver and spleen stiffness measurements.

The relevance of portal hypertension assessment is particularly evident in patients receiving antiviral therapy. Liver stiffness may decline rapidly following viral suppression or eradication, whereas portal hypertension often improves more slowly and may persist despite marked reductions in stiffness. Consequently, longitudinal evaluation of both liver and spleen stiffness, combined with assessment of portal haemodynamics, may provide a more accurate picture of residual risk than liver stiffness alone [15,36,37]. In this setting, MPUS offers a practical, non-invasive framework for monitoring disease evolution and identifying patients who continue to require endoscopic surveillance and specialised follow-up despite successful virological control [12,13,15].

## 9. Steatotic Phenotypes and the Viral–Metabolic Interface

The biological significance of hepatic steatosis in chronic viral hepatitis extends beyond the mere presence of fat within the liver. Rather than representing a uniform entity, steatosis may arise from distinct mechanisms and may have different implications depending on the stage of liver disease, inflammatory activity and the underlying viral infection. In this context, steatosis should be viewed not simply as an imaging finding but as a marker of disease phenotype [17,18,39,40].

In chronic hepatitis C, particularly genotype 3 infection, steatosis may result directly from viral interference with hepatocellular lipid metabolism. Viral proteins affect lipid synthesis, storage and export, promoting triglyceride accumulation within hepatocytes. This virus-associated steatotic phenotype is closely linked to viral replication and inflammatory activity. Accordingly, steatosis may regress rapidly following successful antiviral therapy and may reappear in the setting of virological relapse [39]. In contrast, steatosis observed in non-genotype 3 HCV infection is more often associated with obesity, insulin resistance and other metabolic risk factors, resembling the phenotype observed in MASLD (Table 4) [39,40].

The situation in chronic hepatitis B differs substantially. HBV itself is not generally regarded as a strongly steatogenic virus, and hepatic fat accumulation in HBV-infected individuals is usually attributed to coexisting metabolic dysfunction rather than direct viral effects [40]. Nevertheless, the interaction between viral hepatitis and metabolic liver disease is increasingly recognised. In many patients, chronic HBV infection and metabolic dysfunction coexist and contribute independently to liver injury, fibrosis progression and hepatocarcinogenesis [40].

From an imaging perspective, the increasing overlap between chronic viral hepatitis and metabolic liver disease has important implications. In many contemporary cohorts, metabolic dysfunction is present in a substantial proportion of patients with HBV or HCV infection, and steatosis is no longer a secondary finding but a frequent coexisting component of liver disease [40]. Consequently, interpretation of hepatic fat content requires consideration of the broader clinical context. The presence of steatosis may reflect viral effects, metabolic dysfunction or a combination of both, and its prognostic significance may differ accordingly. This complexity highlights the limitations of interpreting steatosis as a simple binary finding and supports a more phenotype-oriented approach to liver imaging.

Importantly, the relationship between steatosis and liver disease is not static but evolves throughout the natural history of chronic viral hepatitis. In the earlier stages of disease, inflammatory activity frequently dominates the clinical picture. During this phase, steatosis may coexist with necroinflammation and contribute additional metabolic stress to an already injured liver. As fibrosis develops, the relative contribution of inflammation, fibrosis and steatosis changes. The liver progressively transitions from an inflammatory phenotype to a fibrotic phenotype, and eventually, in some patients, to a haemodynamic phenotype characterised by portal hypertension and its complications [15,23,24].

This evolution has important imaging implications. The amount of visible hepatic fat does not necessarily parallel disease severity. Indeed, advanced fibrosis and cirrhosis may be associated with a reduction in hepatic fat content despite ongoing disease progression. Architectural distortion, hepatocellular loss and alterations in lipid metabolism may all contribute to this phenomenon [17,40]. Consequently, the apparent disappearance of steatosis should not be interpreted as evidence of recovery, particularly in patients with advanced fibrosis or portal hypertension.

The concept of changing disease phenotypes is particularly relevant in the era of effective antiviral therapy. Historically, clinicians focused primarily on controlling viral replication and limiting inflammation. Today, many patients achieve long-term viral suppression or complete viral eradication. As the inflammatory component of liver injury diminishes, other drivers of disease become increasingly visible. In some patients, fibrosis and portal hypertension remain the dominant determinants of prognosis [6,15]. In others, metabolic dysfunction emerges as the principal source of ongoing liver injury despite successful viral control [40].

The growing availability of ultrasound-based steatosis quantification methods further supports this approach. Techniques such as controlled attenuation parameter (CAP; Echosens, Paris, France), attenuation imaging (ATI; Canon Medical Systems, Otawara, Japan) and ultrasound-guided attenuation parameter (UGAP; GE HealthCare, Chicago, IL, USA) enable objective estimation of hepatic fat content and facilitate longitudinal monitoring [17,18]. In patients receiving antiviral therapy, repeated quantification of steatosis may be particularly informative because changes in hepatic fat content do not necessarily parallel changes in fibrosis or portal hypertension. While liver stiffness often decreases after successful viral suppression or eradication, metabolic risk factors may persist or even become more clinically relevant over time. Assessment of steatosis therefore contributes not only to disease characterisation but also to the identification of patients in whom metabolic dysfunction remains an important driver of future liver-related risk [17,18,40]. This is particularly relevant when chronic viral hepatitis and metabolic dysfunction coexist. In such patients, attenuation-based quantification provides a repeatable measure of the metabolic component of liver injury that is largely independent of the virological course, and serial measurement after antiviral therapy allows the two trajectories to be followed separately: hepatic fat that regresses after eradication of genotype 3 HCV infection indicates a virus-driven mechanism, whereas fat that persists or increases after viral control identifies patients whose residual risk is metabolic and who require cardiometabolic rather than antiviral management [17,18,39,40]. Acquisition and interpretation of attenuation measurements, including the requirement that values obtained with different systems are not interchangeable and that longitudinal comparisons should be made on the same platform, follow the current WFUMB guidance on liver multiparametric ultrasound [13,14].

From this perspective, antiviral therapy does not simply improve liver disease; it may fundamentally alter its phenotype. A patient initially presenting with active viral hepatitis may subsequently enter a phase dominated by residual fibrosis, portal hypertension or metabolic steatotic liver disease. The challenge for imaging is therefore not only to quantify individual abnormalities but also to characterise how the dominant biological processes evolve over time.

This is one of the areas in which multiparametric ultrasound may provide particular value. Simultaneous assessment of steatosis, liver stiffness, spleen stiffness and portal haemodynamics enables evaluation of several disease domains within a single examination [12,13,14]. Rather than viewing steatosis as an isolated parameter, MPUS allows it to be interpreted within the broader context of inflammation, fibrosis and portal hypertension. Such an integrated approach may prove increasingly important as the population of treated patients continues to grow and the distinction between viral and metabolic liver disease becomes progressively less clear.

## 10. Macrovascular Haemodynamics and Hepatic Perfusion

Progressive fibrosis and cirrhosis induce profound changes in both the macrovascular and microvascular circulation of the liver. While elastography primarily reflects tissue mechanical properties, Doppler ultrasound and contrast-enhanced ultrasound (CEUS) provide insight into the haemodynamic consequences of chronic liver disease. Together, these techniques extend assessment beyond fibrosis and portal pressure surrogates towards direct evaluation of vascular function and perfusion [15,19,23,24].

The clinical value of Doppler ultrasound lies primarily in the assessment of advanced disease. Alterations in portal and hepatic blood flow are generally late manifestations of chronic liver injury and therefore do not play a major role in early fibrosis staging. However, once portal hypertension develops, macrovascular changes become increasingly important because they reflect the physiological consequences of architectural distortion and increased intrahepatic resistance [15,37].

Typical Doppler findings include reduced portal vein velocity, enlargement of the portal and splenic veins, loss of the normal triphasic hepatic venous waveform, increased hepatic arterial flow and, in advanced cases, hepatofugal portal flow. The development of porto-systemic collateral vessels, recanalisation of the paraumbilical vein and splenomegaly provide additional evidence of clinically significant portal hypertension [15,37]. Although none of these findings alone is sufficiently sensitive for early diagnosis, their presence strongly supports advanced disease and may help identify patients at increased risk of decompensation. Taken individually, these measurements have limited sensitivity and specificity. Portal vein diameter and flow velocity in particular are influenced by the respiratory cycle and by the depth of inspiration, by the postprandial state, by cardiac output and right-sided filling pressures, and by technical factors including the angle of insonation, sample-volume placement, transducer pressure and operator experience; reported reproducibility is correspondingly modest. Doppler indices should therefore be interpreted as supportive findings within the examination rather than as independent diagnostic criteria for portal hypertension [15,37].

Importantly, Doppler findings should be interpreted as markers of haemodynamic adaptation rather than fibrosis itself. Their appearance signals a transition from a predominantly structural disease phenotype, characterised by fibrosis, towards a haemodynamic phenotype dominated by portal hypertension and its complications. This distinction is particularly relevant in patients with advanced chronic liver disease, where prognosis is increasingly determined by portal pressure rather than fibrosis stage alone [15,37].

Contrast-enhanced ultrasound extends vascular assessment from the macrocirculation to the microcirculation. By using intravascular microbubble contrast agents, CEUS enables real-time visualisation of hepatic perfusion and contrast transit and is supported by well-established guidelines for hepatic applications [19,29,30]. Beyond lesion characterisation, CEUS offers a unique opportunity to study functional changes in hepatic blood flow associated with chronic liver disease.

One perfusion-based parameter of particular interest is hepatic vein transit time (HVTT), defined as the interval between contrast administration and the appearance of microbubbles within the hepatic veins. Progressive fibrosis and cirrhosis shorten this interval through intrahepatic shunting, vascular remodelling and altered perfusion dynamics. In biopsy-characterised chronic hepatitis C, HVTT has demonstrated high diagnostic accuracy for distinguishing mild disease from cirrhosis [25]. Although limited standardisation and the need for dedicated analysis tools have restricted its widespread clinical adoption, HVTT illustrates an important principle: perfusion may provide biologically meaningful information that is not captured by morphology, stiffness or portal haemodynamics alone.

From a multiparametric perspective, Doppler ultrasound and CEUS complement rather than compete with elastography. Whereas stiffness-based methods estimate structural liver damage and portal hypertension risk, haemodynamic and perfusion imaging provide direct information on vascular adaptation, microcirculatory function and the physiological consequences of chronic liver disease [15,19,23,24,25]. Together, these techniques contribute to a more comprehensive characterisation of the transition from inflammation and fibrosis to portal hypertension and hepatic decompensation.

## 11. Contrast-Enhanced Ultrasound Beyond Perfusion

The most established application of contrast-enhanced ultrasound (CEUS) in chronic viral liver disease is the characterisation of focal liver lesions in patients at risk of hepatocellular carcinoma (HCC). The Contrast-Enhanced Ultrasound Liver Imaging Reporting and Data System (CEUS LI-RADS), developed by the American College of Radiology, standardises terminology, interpretation and reporting of enhancement patterns, allowing liver nodules to be assigned categories reflecting their probability of HCC [29,30]. The characteristic HCC pattern consists of arterial-phase hyperenhancement followed by late and mild washout, a combination associated with high specificity and positive predictive value for the LR-5 category. Sensitivity is lower for very small lesions and for nodules with atypical vascular behaviour [29,30]. CEUS LI-RADS applies only to patients belonging to a defined population at risk of HCC: adults with cirrhosis, adults with chronic hepatitis B who meet surveillance criteria, and patients with current or previously treated HCC. It is not intended for patients outside these categories, including patients with chronic hepatitis C without advanced fibrosis, and it is not applicable to cirrhosis caused by congenital hepatic fibrosis or vascular disorders. Applying LI-RADS categories outside the at-risk population, where the pre-test probability of HCC is low, risks misclassification of benign lesions such as haemangioma or focal nodular hyperplasia [29,30].

Importantly, CEUS should not be regarded as a primary surveillance tool for HCC. Current surveillance strategies continue to rely on conventional B-mode ultrasound, with CEUS reserved for the characterisation of lesions detected during screening or identified by other imaging modalities [19,29,30]. Although CEUS may substantially improve diagnostic confidence and reduce the need for additional imaging in selected patients, its role in population-level surveillance remains insufficiently validated. As a result, current international guidelines position CEUS as a problem-solving and lesion-characterisation tool rather than a first-line surveillance modality [19,29,30].

In chronic viral hepatitis, this capability is valuable at several points in the diagnostic pathway. CEUS provides a real-time, radiation-free means of characterising indeterminate nodules detected during routine surveillance and may allow for definitive assessment during the same examination. This is particularly relevant in the growing population of patients who remain at risk of HCC despite successful antiviral therapy. In these individuals, surveillance frequently continues for many years after viral suppression or eradication because the risk is determined primarily by pre-existing fibrosis, cirrhosis and portal hypertension rather than by viral replication itself [6,7,8].

Beyond lesion characterisation, CEUS offers insight into tumour biology, hepatic microcirculation and reticuloendothelial function. Most currently used ultrasound contrast agents remain confined to the vascular compartment and therefore primarily depict vascular architecture and perfusion dynamics [19]. However, not all contrast agents behave identically. Sonazoid (perfluorobutane microbubbles; GE HealthCare, Oslo, Norway) is unique in that it is phagocytosed by Kupffer cells, the resident macrophages of the liver. This property permits imaging during a delayed Kupffer phase, reflecting not only perfusion but also the integrity of the hepatic reticuloendothelial system [41,42].

The biological implications of Kupffer-phase imaging are particularly intriguing in chronic liver disease. Progressive fibrosis, chronic inflammation and hepatocarcinogenesis are accompanied by alterations in Kupffer cell density and function. Most HCCs contain fewer functioning Kupffer cells than the surrounding liver parenchyma and therefore appear as focal defects during the Kupffer phase [41,42]. Consequently, Sonazoid-enhanced imaging provides information complementary to conventional vascular-phase assessment and may improve lesion detection, particularly in small or otherwise inconspicuous tumours.

At present, however, Kupffer-phase imaging remains less extensively validated than conventional CEUS approaches based on vascular enhancement patterns. Its clinical adoption varies considerably between regions, and further studies are required to determine its precise role within surveillance and diagnostic algorithms [41,42]. Nevertheless, the ability to interrogate both vascular perfusion and reticuloendothelial function illustrates how CEUS may evolve beyond simple lesion characterisation towards a more comprehensive biological assessment of chronic liver disease.

From a multiparametric perspective, CEUS complements elastography and haemodynamic assessment by addressing a different dimension of liver pathology: focal neoplasia, tissue perfusion and reticuloendothelial function. For patients with chronic viral hepatitis, particularly those with advanced fibrosis or cirrhosis, this integration is attractive because it allows risk assessment, evaluation of portal hypertension and lesion characterisation to be performed within a single imaging platform [13,15,19]. More broadly, CEUS exemplifies the evolution of liver ultrasound from morphology-based imaging towards functional and biological characterisation of disease. Its greatest value in the elimination era may therefore lie not only in diagnosing HCC, but also in supporting long-term surveillance of the growing population of treated patients who remain at residual oncological risk [7,8,41,42].

An important biological distinction applies to chronic hepatitis B in this context. After HCV cure, the residual risk of HCC is concentrated in patients with advanced fibrosis or cirrhosis, and surveillance recommendations follow the baseline fibrosis stage. In chronic hepatitis B, hepatocarcinogenesis may occur in a non-cirrhotic liver, because integration of viral DNA into the host genome, expression of HBx and other virus-related oncogenic mechanisms operate independently of the fibrogenic pathway; surveillance is therefore recommended in defined non-cirrhotic HBV populations on the basis of age, sex, family history, ethnic background, viral load and validated risk scores rather than on the basis of stiffness alone [5,43,44]. Consequently, a fall in liver stiffness during nucleos(t)ide analogue therapy, or a value below the conventional thresholds for advanced fibrosis, does not justify discontinuing HCC surveillance in patients who meet these criteria. In both infections, the decision to continue or discontinue surveillance follows current EASL and AASLD recommendations and is not taken on the basis of ultrasound-derived parameters alone [43,44].

## 12. Beyond Stiffness: Emerging Biomarkers of Liver Tissue Biology

The evolution of multiparametric ultrasound has progressively shifted liver imaging from the assessment of morphology towards the characterisation of tissue biology. Elastography, attenuation imaging, Doppler ultrasound and CEUS already provide information on fibrosis, steatosis, portal hypertension and perfusion. Nevertheless, many of the biological processes that drive disease progression begin before advanced fibrosis develops and remain only partially captured by currently established biomarkers. This has stimulated interest in a new generation of ultrasound techniques designed to probe microvascular function, tissue composition and biomechanical properties at a more fundamental level.

A common theme underlying these approaches is the recognition that fibrosis represents a relatively late manifestation of chronic liver injury. Long before cirrhosis becomes apparent, the liver undergoes inflammatory activation, sinusoidal endothelial dysfunction, microvascular remodelling, extracellular matrix reorganisation and changes in tissue composition [23,24]. Emerging ultrasound biomarkers attempt to detect these processes directly rather than infer them indirectly from liver stiffness.

### 12.1. Microvascular Imaging

High-sensitivity Doppler techniques, including superb microvascular imaging (SMI; Canon Medical Systems, Otawara, Japan) and related microvascular flow imaging methods, allow for visualisation of low-velocity blood flow within vessels that are below the detection threshold of conventional colour Doppler. These techniques have generated considerable interest because microvascular remodelling is a central feature of chronic viral hepatitis and fibrogenesis [23,24,26,27]. These methods display a Doppler signal arising from low-velocity flow; they do not measure microvascular remodelling or sinusoidal capillarisation directly, and the relationship between the displayed vascular pattern and the underlying histological changes remains inferential.

In chronic hepatitis B, SMI-derived vascular scores have demonstrated correlations with histological fibrosis stage and inflammatory activity. Some studies have reported diagnostic performance comparable to, or even exceeding, shear-wave elastography for fibrosis classification [26]. Similar observations have been made in chronic hepatitis C, where progressive alterations in the visualised vascular architecture correlate with fibrosis severity and histological markers of sinusoidal capillarisation [27]. These studies are small, single-centre and vendor-specific; were not designed to test clinical outcomes; and the vascular scores reported are not interchangeable between systems.

Although these findings remain preliminary, they are biologically plausible. The transition from a fenestrated sinusoidal network to a capillarised microvascular bed is a key event in chronic liver disease and may precede the development of advanced fibrosis [23,24]. Techniques sensitive to these changes might therefore identify biologically active disease before conventional markers become abnormal. This remains a hypothesis: no standardised acquisition protocol, reference range or outcome-validated threshold is currently available, and the biological interpretation of these signals has not been confirmed against histology in prospective cohorts.

### 12.2. Viscosity Imaging and Shear-Wave Dispersion

Shear-wave elastography primarily reflects tissue elasticity. However, biological tissues also possess viscous properties that influence the propagation of mechanical waves. Shear-wave dispersion imaging exploits this phenomenon by measuring the frequency dependence of shear-wave velocity, providing an indirect estimate of tissue viscosity [28].

From a biological perspective, viscosity may be more closely related to inflammatory activity, tissue fluid content and cellular injury than to fibrosis itself. Dispersion-based measurements should nevertheless be understood as an estimate of a mechanical property of tissue, influenced by fluid content, congestion, temperature and vendor-specific processing, rather than as a direct measurement of inflammatory activity. This is particularly attractive in chronic viral hepatitis, where necroinflammation represents the major confounder of stiffness measurements. In theory, combining stiffness and viscosity measurements could help separate the fibrotic and inflammatory components of chronic liver disease.

Early studies have demonstrated associations between dispersion slope measurements and biochemical or histological markers of inflammation [28]. However, standardisation remains incomplete, normal ranges are not firmly established and the incremental value of dispersion imaging beyond conventional elastography has yet to be consistently demonstrated. At present, viscosity imaging should therefore be regarded as a promising research tool rather than a mature clinical biomarker. Dispersion values are additionally vendor-dependent and cannot be transferred between systems, and no threshold has been validated against clinical outcomes.

### 12.3. Quantitative Ultrasound and Tissue Characterisation

A particularly active area of research involves quantitative ultrasound (QUS), which seeks to extract information from raw radiofrequency (RF) ultrasound signals rather than relying solely on conventional greyscale images. Unlike elastography, which measures a mechanical property, QUS attempts to characterise the acoustic behaviour of tissue itself.

Several approaches have been explored, including backscatter coefficient analysis, spectral analysis, envelope statistics, entropy imaging, texture analysis and Nakagami imaging. These methods aim to quantify microscopic tissue organisation, cellularity and structural heterogeneity. In principle, they may provide information regarding fibrosis, inflammation, steatosis and architectural remodelling simultaneously.

Backscatter-based techniques are of particular interest because extracellular matrix deposition, collagen organisation and changes in hepatic microarchitecture all influence acoustic scattering. Likewise, statistical approaches such as Nakagami imaging may reflect alterations in tissue complexity that occur during progressive fibrosis. Preliminary studies suggest that some QUS parameters correlate with histological fibrosis stage and may detect subtle changes not captured by conventional B-mode imaging.

Despite these promising results, evidence specific to chronic viral hepatitis remains limited. Most studies are single-centre investigations employing different acquisition protocols and analysis methods, making direct comparison difficult. Consequently, QUS remains largely confined to research settings. Quantitative ultrasound parameters likewise describe acoustic properties of tissue rather than specific biological processes, and their attribution to fibrosis, inflammation or microstructural remodelling remains provisional.

### 12.4. The Biological Target: Endothelial Dysfunction and Microvascular Remodelling

Although technically diverse, many emerging ultrasound biomarkers converge on a common biological target: the hepatic microcirculation. Liver sinusoidal endothelial cells (LSECs) maintain the specialised low-resistance vascular environment of the healthy liver and regulate vascular tone, immune signalling and interactions with hepatic stellate cells [23].

**Figure 6:**
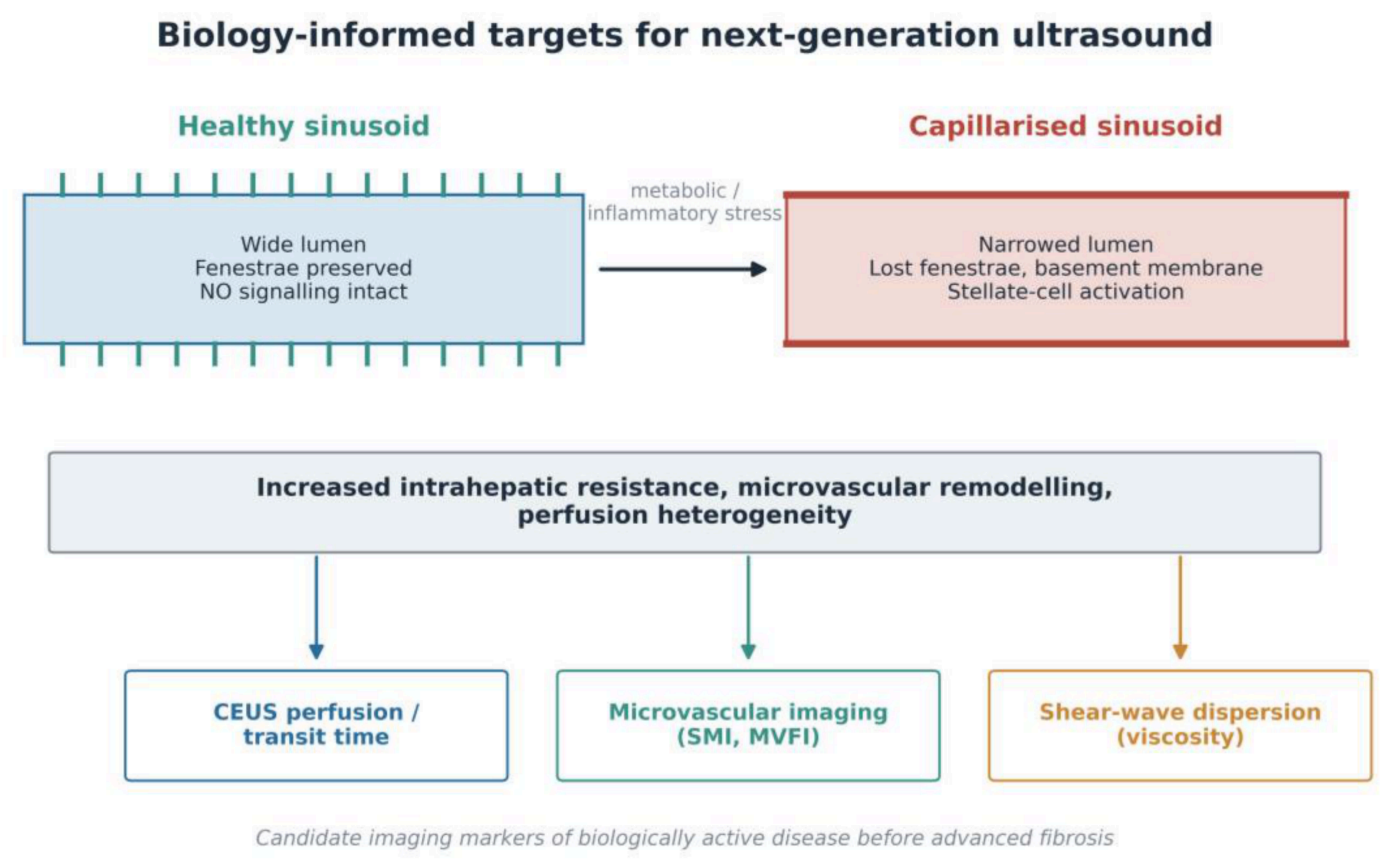
Biology-informed targets for next-generation ultrasound. Liver sinusoidal endothelial cell dysfunction, capillarisation, increased intrahepatic vascular resistance and microvascular remodelling precede advanced fibrosis and provide the biological substrate that perfusion, microvascular and viscosity imaging aim to detect, framing multiparametric ultrasound as an examination-level, biology-informed strategy rather than a set of competing measurements.

Chronic viral hepatitis disrupts this homeostatic system. Persistent inflammatory injury promotes loss of sinusoidal fenestrae, basement membrane formation, impaired nitric oxide signalling and progressive sinusoidal capillarisation [23,24]. These alterations increase intrahepatic vascular resistance, contribute to portal hypertension and facilitate activation of hepatic stellate cells, thereby linking inflammation, haemodynamic dysfunction and fibrosis. Importantly, many of these changes occur before advanced fibrosis becomes histologically apparent [23,24]. LSEC alterations have been documented directly in patients with chronic hepatitis C and are increasingly recognised as central drivers of disease progression (Figure 6) [24].

This biological framework provides a rationale for the development of microvascular imaging, viscosity-sensitive techniques and quantitative tissue characterisation. Rather than measuring fibrosis alone, these approaches seek to interrogate the upstream processes that ultimately lead to fibrosis and portal hypertension. Whether they will eventually improve clinical decision-making remains uncertain. However, they collectively point towards a future in which liver ultrasound moves beyond structural assessment and increasingly functions as a tool for non-invasive biological phenotyping of chronic liver disease [23,24,26,27,28]. It should be emphasised that none of these techniques currently measures endothelial dysfunction, microvascular remodelling or inflammatory activity directly. Each provides a physical measurement whose biological interpretation is inferred and remains to be validated, and none is at present suitable for use in clinical decision-making.

## 13. From Structural Imaging to Biological Phenotyping

The evolution of liver ultrasound mirrors the changing landscape of chronic viral hepatitis. Historically, imaging focused on detecting the structural consequences of disease, including fibrosis, cirrhosis and portal hypertension. The development of elastography, attenuation imaging, Doppler ultrasound and contrast-enhanced ultrasound has substantially improved non-invasive assessment, reducing reliance on liver biopsy and enabling longitudinal monitoring of large patient populations [9,12,13,14,19].

Nevertheless, important challenges remain. Most established imaging biomarkers primarily reflect the downstream consequences of chronic liver injury rather than the biological mechanisms that drive disease progression. Fibrosis, steatosis and portal hypertension are clinically important endpoints, but they represent different stages of a complex process involving inflammation, sinusoidal endothelial dysfunction, microvascular remodelling and alterations in tissue composition [23,24].

This distinction is particularly relevant after successful antiviral therapy, when improvement in liver stiffness and biochemical markers may coexist with residual fibrosis, portal hypertension and persistent risk of hepatocellular carcinoma [6,7,8,35,36,45]. The central challenge is therefore no longer simply to detect advanced liver disease, but to identify patients in whom biologically active disease processes continue despite apparent clinical improvement.

In this context, multiparametric ultrasound is best viewed not as a collection of competing technologies but as an integrated, examination-level strategy for biological phenotyping, in which value lies in the combination of complementary parameters within a single examination rather than in any individual measurement [12,13,14].

Elastography provides information on tissue mechanics, attenuation-based methods assess steatosis, Doppler ultrasound evaluates haemodynamic adaptation, CEUS characterises perfusion and focal lesions, while emerging techniques increasingly target microvascular function and tissue composition [12,13,15,19,23,24,26,27,28].

An important consequence of this conceptual shift is that no single parameter can adequately characterise chronic liver disease. Liver stiffness, steatosis, portal haemodynamics and perfusion each describe different aspects of the disease process and may evolve independently over time. A patient may demonstrate substantial improvement in liver stiffness after antiviral therapy while continuing to exhibit clinically significant portal hypertension or persistent metabolic liver injury [15,16,35,36,37]. Conversely, stable fibrosis may coexist with ongoing inflammatory or microvascular activity [20,21,22,23,24,28]. Interpretation of these findings therefore requires integration rather than reliance on individual biomarkers.

The increasing availability of quantitative ultrasound techniques also raises the possibility of moving beyond stage-based assessment towards continuous risk estimation. Rather than classifying patients solely according to fibrosis stage or the presence of cirrhosis, future multiparametric approaches may provide composite biological profiles incorporating structural, haemodynamic, metabolic and microvascular information. Such models could better reflect the heterogeneity of chronic viral hepatitis and may prove particularly valuable in the growing population of treated patients, where traditional staging systems often fail to capture residual risk adequately [12,23,24,26,27,28,40].

A further challenge will be the shift from parameter-based towards examination-level interpretation: current algorithms generally use individual biomarkers in isolation, whereas the future development of MPUS may depend less on individual technologies than on integrating complementary measurements into clinically meaningful risk models [15,23,24,26,27,28].

## 14. Limitations

Several limitations should be acknowledged. As a narrative rather than systematic review, this article did not follow a predefined protocol, formal risk-of-bias appraisal or quantitative synthesis, and the selection of literature was inevitably influenced by the authors’ judgement. Much of the evidence for emerging techniques, including microvascular imaging, viscosity-sensitive methods and quantitative ultrasound, derives from single-centre studies with heterogeneous acquisition protocols and limited external validation, so the diagnostic-accuracy estimates cited here should be regarded as indicative rather than definitive. Many established thresholds were derived in specific populations and may not transfer directly to treated or comorbid patients, and few studies have evaluated multiparametric ultrasound as an integrated protocol against clinically meaningful long-term outcomes. Prospective, multicentre studies with standardised acquisition and hard clinical endpoints are therefore needed before an integrated multiparametric approach can be recommended for routine risk stratification. In addition, the categories used in Table 1 and Table 5 to describe the level of clinical adoption reflect a pragmatic judgement by the authors rather than a formal evidence-grading system, and the clinical images reproduced here are single illustrative cases from our own practice, intended to demonstrate imaging appearances rather than to provide evidence of diagnostic performance.

## 15. Conclusions

The management of chronic viral hepatitis is undergoing a profound transformation. The widespread implementation of highly effective antiviral therapies has shifted the focus of clinical care from controlling viral replication alone to the long-term management of a growing population of patients living with treated HBV infection or cured HCV infection [4,5]. In this new landscape, the major clinical challenge is no longer simply the detection of advanced fibrosis but the identification and monitoring of residual liver disease, persistent portal hypertension and ongoing risk of hepatocellular carcinoma [6,15].

Multiparametric ultrasound is particularly well suited to address these evolving needs. By integrating conventional B-mode imaging, liver and spleen stiffness assessment, steatosis quantification, Doppler haemodynamic evaluation and contrast-enhanced ultrasound within a single examination, MPUS provides a comprehensive and repeatable assessment of chronic liver disease [12,13,14,19]. Importantly, its value extends beyond diagnosis. The ability to establish a robust baseline before antiviral therapy and to track changes in liver stiffness, portal haemodynamics, steatosis and focal lesions over time makes MPUS a practical tool for longitudinal patient management [15,16,35,36].

Baseline evaluation of liver stiffness, spleen stiffness, portal haemodynamics and steatotic burden before antiviral therapy provides the reference framework required for interpreting subsequent changes; without it, residual portal hypertension may be underestimated [15,16,35,36,45].

In the era of highly effective antiviral therapy, the principal role of MPUS may be shifting from cross-sectional staging towards longitudinal disease profiling. Whenever feasible and clinically indicated, a baseline assessment before antiviral therapy facilitates interpretation of subsequent changes, although a comprehensive multiparametric examination should not be regarded as a precondition for initiating antiviral treatment.

Throughout the natural history of chronic viral hepatitis, different biological processes dominate disease progression. Inflammatory activity characterises earlier stages of disease, fibrosis and architectural remodelling emerge over time, and portal hypertension increasingly determines clinical outcomes in advanced disease [15,23,24]. Following successful antiviral therapy, these processes may evolve independently: inflammation often resolves rapidly, liver stiffness may decline, while portal hypertension, microvascular remodelling and hepatocellular carcinoma risk can persist for years [6,15,16,35,36,45].

The established components of MPUS—including elastographic fibrosis staging, Baveno VII liver and spleen stiffness criteria for clinically significant portal hypertension, attenuation-based quantification of steatosis, Doppler assessment of portal haemodynamics and CEUS characterisation of focal liver lesions—already provide a robust framework for non-invasive monitoring [13,14,15,16,19,29,30]. Emerging techniques such as microvascular imaging, viscosity-sensitive methods and quantitative ultrasound may further expand this framework by targeting endothelial dysfunction, microvascular remodelling and tissue composition [23,24,26,27,28,46,47,48,49]. However, their clinical utility remains to be confirmed through prospective validation studies using clinically meaningful endpoints.

The history of imaging in chronic viral hepatitis has largely been the history of fibrosis assessment. The future may be different. As viral hepatitis moves towards elimination, the principal challenge shifts from detecting structural liver damage to understanding residual disease biology and predicting future risk [2,3].

From diagnosis and baseline staging, through treatment monitoring, to long-term surveillance for portal hypertension and hepatocellular carcinoma, MPUS has the potential to accompany patients throughout the entire course of disease (Table 5). If future studies demonstrate that integrated multiparametric assessment improves prediction of clinically meaningful outcomes, multiparametric ultrasound may evolve from a tool for measuring fibrosis towards a potential, still investigational framework for personalised, biology-informed and non-invasive phenotyping of chronic liver disease in the era of viral hepatitis elimination efforts [7,8,12,15,23,24,26,27,28,37,40,41,42].

## Figures and Tables

**Table 1:** Multiparametric ultrasound components mapped to biological target, principal clinical question in chronic viral hepatitis, and current level of clinical adoption.

Modality	Biological Target	Principal Clinical Question	Level of Clinical Adoption
B-mode ultrasound	Morphology, surface, vasculature	Cirrhosis morphology, nodules, surveillance entry	Guideline-endorsed
Shear-wave elastography (TE, pSWE, 2D-SWE)	Matrix deposition, tissue stiffness	Fibrosis staging and longitudinal monitoring	Guideline-endorsed
Liver and spleen stiffness (Baveno VII)	Intrahepatic resistance, portal pressure	Clinically significant portal hypertension	Guideline-endorsed
Attenuation (CAP, ATI, UGAP)	Hepatic triglyceride content	Steatosis grade; viral–metabolic overlap	Guideline-endorsed (CAP); emerging (ATI/UGAP)
CEUS LI-RADS	Lesion vascularity	HCC nodule characterisation (problem-solving; not surveillance)	Guideline-endorsed
Doppler/CEUS perfusion (HVTT)	Macro- and microvascular haemodynamics	Portal hypertension; functional severity	Emerging
Shear-wave dispersion/viscosity	Inflammation, tissue fluid state	Necroinflammatory activity	Investigational
Microvascular imaging (SMI, MVFI)	Microvascular remodelling, capillarisation	Biologically active disease	Investigational
Quantitative tissue characterisation	Microstructural organisation	Tissue heterogeneity	Investigational

TE, transient elastography; pSWE, point shear-wave elastography; 2D-SWE, two-dimensional shear-wave elastography; CAP, controlled attenuation parameter; ATI, attenuation imaging; UGAP, ultrasound-guided attenuation parameter; CEUS, contrast-enhanced ultrasound; LI-RADS, Liver Imaging Reporting and Data System; HVTT, hepatic vein transit time; SMI, superb microvascular imaging; MVFI, microvascular flow imaging; HCC, hepatocellular carcinoma. The categories in the final column describe the current level of clinical adoption as judged by the authors, on the basis of inclusion in current EFSUMB, WFUMB, Baveno VII and CEUS LI-RADS documents [9,10,13,14,15,19,29,30]: guideline-endorsed, recommended for a defined clinical question in at least one current international document; emerging, supported by peer-reviewed clinical data but not yet incorporated into guideline recommendations; investigational, confined to research settings, without standardised acquisition, reference ranges or outcome-validated thresholds. This is a pragmatic categorisation of clinical adoption and not a formal evidence-grading system such as GRADE.

**Table 2:** Representative diagnostic performance of elastography for fibrosis staging in chronic viral hepatitis (illustrative, derived from cited cohorts and meta-analyses; values are not directly comparable between rows; thresholds are activity-dependent).

Technique/Population	Target	AUROC (Approx.)	Illustrative Cut-Off
TE, chronic hepatitis B [34]	Cirrhosis (F4)	0.93	≈11.7 kPa
TE, chronic hepatitis B [34]	Significant fibrosis (F2)	0.86	≈7.9 kPa
2D-SWE, chronic hepatitis C [31]	Cirrhosis	≈0.93	Etiology-dependent
2D-SWE, chronic hepatitis B [31]	Cirrhosis	≈0.96	Etiology-dependent
pSWE, viral hepatitis (pooled) [32,33]	Cirrhosis	≈0.91	Etiology-dependent
pSWE, viral hepatitis (pooled) [32,33]	Significant fibrosis	≈0.84	Etiology-dependent

AUROC, area under the receiver-operating-characteristic curve; TE, transient elastography; 2D-SWE, two-dimensional shear-wave elastography; pSWE, point shear-wave elastography. Values are approximate and should not be applied during active necroinflammation without activity-adjusted thresholds. The estimates listed here originate from different meta-analyses and cohorts, which differ in reference standard (METAVIR or Ishak staging on biopsies of variable length and quality), in the prevalence and spectrum of fibrosis stages, in the elastography systems and acquisition protocols used, and in the method by which cut-offs were derived. The table is therefore a descriptive summary of reported performance and does not represent a head-to-head comparison; differences between rows should not be interpreted as evidence that one technique outperforms another.

**Table 3:** Baveno VII stiffness-based rules for clinically significant portal hypertension applied to viral compensated advanced chronic liver disease.

Parameter	Rule-Out CSPH	Rule-In CSPH
Liver stiffness (VCTE)	≤15 kPa with platelets ≥ 150 × 10^9^/L	≥25 kPa
Spleen stiffness (VCTE)	<21 kPa	>50 kPa
Indeterminate zone	Liver stiffness 15–25 kPa; refine with spleen stiffness	Combine liver + spleen stiffness; consider HVPG/endoscopy

CSPH, clinically significant portal hypertension; VCTE, vibration-controlled transient elastography; HVPG, hepatic venous pressure gradient. VCTE thresholds should not be applied directly to point or two-dimensional shear-wave elastography, and spleen-stiffness thresholds additionally depend on technique, vibration frequency and population. Thresholds per the Baveno VII consensus [15,16]; spleen-stiffness thresholds apply to cACLD due to viral hepatitis.

**Table 4:** Origins and imaging behaviour of hepatic steatosis in chronic viral hepatitis.

Context	Dominant Mechanism	Behaviour After Viral Control
HCV genotype 3	Virus-induced (cytopathic) steatosis via core-protein effects on lipid metabolism	Typically regresses with SVR; recurs with relapse
HCV non-genotype 3	Predominantly metabolic/host factors	Persists if metabolic drivers continue
Chronic hepatitis B	Usually coexisting metabolic dysfunction (MASLD overlap)	Persists or progresses independent of viral suppression

HCV, hepatitis C virus; SVR, sustained virological response; MASLD, metabolic dysfunction-associated steatotic liver disease [17,18,39].

**Table 5:** Elimination-era clinical questions mapped to the multiparametric ultrasound examination.

Clinical Question	Primary MPUS Parameter(s)	Supporting Evidence
Baseline fibrosis stage	Shear-wave elastography	Guideline-endorsed
Fibrosis/inflammation after therapy	Serial stiffness with viscosity context	Guideline-endorsed (stiffness); investigational (viscosity)
Clinically significant portal hypertension	Liver + spleen stiffness (Baveno VII)	Guideline-endorsed
Steatosis and viral–metabolic overlap	Attenuation (CAP/ATI/UGAP)	Guideline-endorsed (CAP)
HCC surveillance and nodule characterisation	Surveillance: B-mode US (with or without AFP); nodule characterisation: CEUS LI-RADS or multiphasic CT/MRI	Guideline-endorsed

MPUS, multiparametric ultrasound; CAP, controlled attenuation parameter; ATI, attenuation imaging; UGAP, ultrasound-guided attenuation parameter; CEUS, contrast-enhanced ultrasound; LI-RADS, Liver Imaging Reporting and Data System; HCC, hepatocellular carcinoma. The categories in the final column follow the level-of-clinical-adoption scheme defined in the footnote to Table 1.

## Data Availability

No new data were created or analysed in this study. Data sharing is not applicable to this article.
